# Supplementary material for: Inhibition of BRD4 Reduces Neutrophil Activation and Adhesion to the Vascular Endothelium Following Ischemia Reperfusion Injury
Source: Int J Mol Sci. 2020 Dec 17;21(24):9620. doi: 10.3390/ijms21249620 (PMC7767067; doi:10.3390/ijms21249620)
Supplement: Supplementary file 1 [file ijms-21-09620-s001.pdf]

Inhibition of BRD4 reduces neutrophil activation and adhesion to the vascular endothelium following ischemia reperfusion injury

Shelby Reid*[1], Noah Fine[2], Vikrant K. Bhosle[3], Joyce Zhou[1], Rohan John[4], Michael Glogauer[2,5,6], Lisa A. Robinson, [1,3,7,8], James W. Scholey[1,9]

Genes	4 Hours (q-value)	24 Hours q-value	48 Hours q-value
Junb	logFC = ****	logFC = ****	logFC = ****
Rel	logFC = 0.9992	logFC = 0.9996	logFC = 0.9901
Il6	logFC = 0.9992	logFC = 0.9996	logFC = 0.9901
Cxcl1	logFC = ****	logFC = 0.0774	logFC = **
Plaur	logFC = *	logFC = ****	logFC = ****
Tnfaip3	logFC = 0.9992	logFC = 0.9996	logFC = 0.9901
Myc	logFC = **	logFC = ***	logFC = *
Orm2	logFC = 0.9992	logFC = 0.9996	logFC = 0.9901
Il11	logFC = 0.9992	logFC = 0.7555	logFC = 0.9901
Brca1	logFC = 0.9992	logFC = 0.9996	logFC = 0.9901
Xdh	logFC = 0.9992	logFC = ****	logFC = **
Vim	logFC = 0.9992	logFC = ****	logFC = ****
Il1rn	logFC = 0.9992	logFC = 0.9996	logFC = 0.9901
Lbp	logFC = 0.9992	logFC = 0.9996	logFC = 0.4402
Mmp9	logFC = 0.9992	logFC = 0.9996	logFC = 0.9761
Csf1	logFC = 0.9992	logFC = *	logFC = *
Penk	logFC = 0.9992	logFC = 0.9996	logFC = 0.9761
Tnc	logFC = 0.9992	logFC = **	logFC = *
Cxcl10	logFC = 0.5823	logFC = 0.8058	logFC = 0.7971
Icam1	logFC = 0.5823	logFC = *	logFC = 0.0795
Lgals3	logFC = ****	logFC = ****	logFC = ****
Bgn	logFC = 0.2233	logFC = ****	logFC = ****
Igfbp2	logFC = 0.9992	logFC = 0.9996	logFC = 0.9901
Il1b	logFC = 0.9992	logFC = 0.9996	logFC = 0.9901
Vcam1	logFC = 0.9992	logFC = 0.6248	logFC 0.4354
Ptafr	logFC = 0.9992	logFC = 0.9996	logFC = 0.9901
Ccl5	logFC = 0.9992	logFC = 0.9996	logFC = 0.9901
Ccl3	logFC = 0.9992	logFC = 0.9996	logFC = 0.9901
C4b	logFC = 0.9992	logFC = 0.9996	logFC = 0.9901
Ltb	logFC = 0.9992	logFC = 0.9996	logFC = 0.9901
Apoc3	logFC = 0.9992	logFC = 0.0775	logFC = *
Hmox1	logFC = ****	logFC = ****	logFC = ****
Ccl2	logFC = 0.9992	logFC = 0.9996	logFC 0.4354
Ccr5	logFC = 0.9992	logFC = 0.9996	logFC = 0.9901

Table S1. NFκB-mediated genes following IRI, as identified in Figure 1, with their respective fold change. *$p<0.05$, **$p<0.01$, ***$p<0.001$, ****$p<0.0001$.

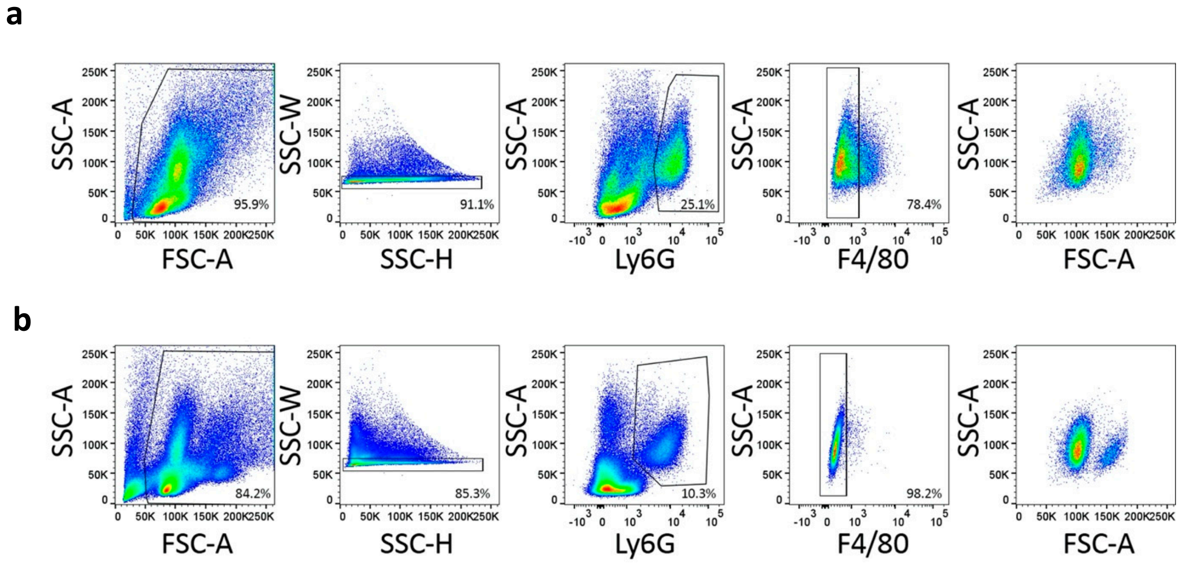

Figure **S1.** Flow cytometry gating strategy for bone marrow **(a)** and blood **(b)**. Mouse PMNs were gated based on Ly6G^{+ve}/F4/80^{-ve}. Doublets were excluded using SSC-W x SSC-H.

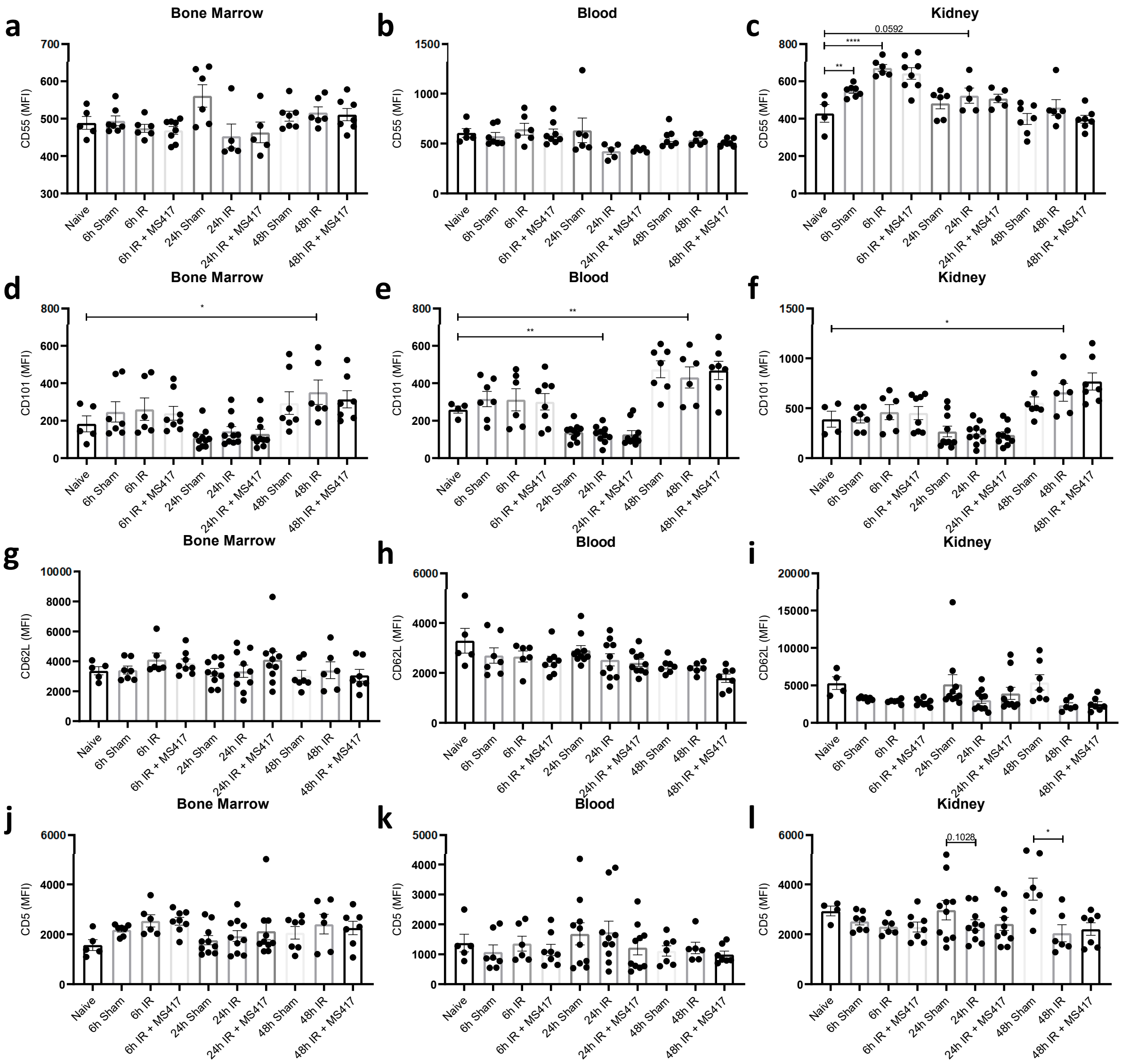

Figure S2. BRD4 inhibition had no effect on CD55 **(a-c)**, CD101 **(d-f)**, CD62L **(g-i)** or CD5 **(j-l)** expression on neutrophils in the bone marrow, blood, or kidney tissue following IRI. C57BL/6 mice were treated with 1μM MS417 daily for 7 days by oral gavage before unilateral IRI. Bone marrow, blood, and kidney tissue was collected at 6, 24, and 48 hours following IRI and neutrophils were quantified by flow cytometry. MFI = mean fluorescence intensity. ANOVA with Fischers LSD test was performed. n = 7-11, *p<0.05, **p<0.01, ***p<0.001, ****p<0.0001.
